# Folate-producing bifidobacteria: metabolism, genetics, and relevance

**DOI:** 10.20517/mrr.2023.59

**Published:** 2023-12-12

**Authors:** Maria Rosaria D’Aimmo, Maria Satti, Donatella Scarafile, Monica Modesto, Stefano Pascarelli, Simone Andrea Biagini, Donata Luiselli, Paola Mattarelli, Thomas Andlid

**Affiliations:** ^1^BioGaia AB, Bioventure Hub, HE424, c/o AstraZeneca AB, R&D, Mölndal 43150, Sweden.; ^2^Department of Agricultural and Food Sciences, University of Bologna, Bologna 40127, Italy.; ^3^Protein Engineering and Evolution Unit, Okinawa Institute of Science, Technology Graduate University, Okinawa 40-0193, Japan.; ^4^Institut de Biologia Evolutiva (UPF-CSIC), Departament de Medicina i Ciències de la Vida, Universitat Pompeu Fabra, Parc de Recerca Biomèdica de Barcelona, Barcelona 08003, Spain.; ^5^Department for the Cultural Heritage (DBC), University of Bologna, Ravenna 48121, Italy.; ^6^Andlid Bio Solutions AB, Gothenburg 41871, Sweden.

**Keywords:** Bifidobacteria, folate metabolism, folate biofortification, gut microbiota

## Abstract

Folate (the general term for all bioactive forms of vitamin B_9_) plays a crucial role in the evolutionary highly conserved one-carbon (1C) metabolism, a network including central reactions such as DNA and protein synthesis and methylation of macromolecules. Folate delivers 1C units, such as methyl and formyl, between reactants. Plants, algae, fungi, and many bacteria can naturally produce folate, whereas animals, including humans, must obtain folate from external sources. For humans, folate deficiency is, however, a widespread problem. Bifidobacteria constitute an important component of human and many animal microbiomes, providing various health advantages to the host, such as producing folate. This review focuses on bifidobacteria and folate metabolism and the current knowledge of the distribution of genes needed for complete folate biosynthesis across different bifidobacterial species. Biotechnologies based on folate-trophic probiotics aim to create fermented products enriched with folate or design probiotic supplements that can synthesize folate in the colon, improving overall health. Therefore, bifidobacteria (alone or in association with other microorganisms) may, in the future, contribute to reducing widespread folate deficiencies prevalent among vulnerable human population groups, such as older people, women at child-birth age, and people in low-income countries.

## INTRODUCTION

Folate, also known as vitamin B_9_, belongs to the water-soluble B-vitamin family. The IUPAC-IUB Joint Commission on Biochemical Nomenclature (JCBN)^[1]^ suggested the use of the term folate for each member of the family of pteroylglutamates or a combination of them, with different levels of oxidation of the pteridine ring, one-carbon substitutions and numbers of glutamate units. Folic acid, in particular, should only be used when referring to the synthetic form of vitamin B_9_^[2]^.

Folate is an essential vitamin implicated in pivotal metabolic pathways. Without folate, cells cannot properly synthesize or methylate DNA and proteins. Many microorganisms and plants can obtain folate *de novo*, whereas vertebrates and other animals, such as insects, need to obtain folate via nutritional sources^[3]^. Understanding that sufficient folate intake reduces the risk for neural tube defects (NTD) is considered one of the most important health-related discoveries ever made^[4]^. Folate deficiency, instead, has been linked to several health disorders, such as coronary heart disease, osteoporosis, Alzheime’rs disease, and colorectal cancer^[5]^. Moreover, folate deficiency causes anemia because it is required for red blood cell production. In general, folate requirement is higher during rapid cell division and growth periods, such as throughout infancy and pregnancy^[6]^.

Folate naturally occurs in leafy green vegetables, oranges, beans, rice, and liver. Natural forms of folates can also be produced by many microorganisms commonly present in the gastrointestinal microbiota, such as bifidobacteria, lactobacilli, and yeasts^[7]^. These forms of folate can be absorbed and utilized by animal or human hosts. Folate is absorbed via transporters in the gastrointestinal epithelia, and through the circulatory system, it then reaches the liver, where it may be stored. The human diet varies in folate content and bioavailability, and there is a substantial loss during processing, storage, and cooking. Therefore, the suggested targets for daily folate intake are often uncertain.

Nevertheless, the World Health Organization recommends that pregnant women adhere to a healthy, balanced diet and take 0.4 mg of the folic acid supplement daily to ensure a healthy pregnancy and improve pregnancy outcomes^[8]^. For this reason, several health authorities have proposed mandatory food fortification with synthetic folic acid. For example, when used as a fortificant in cereals and bread, folic acid can be considered cost-efficient in production, more stable than natural food folate, and superior in terms of bioavailability and bioefficacy. In Australia, food fortification has been implemented since 2009, and in the USA and Canada since 1998, and in both cases, rates of incidence of NTDs have dramatically declined^[6,9]^. Nevertheless, fortification is questioned because a high synthetic folic acid intake could be involved in promoting subclinical cancers and other adverse health effects^[10]^. For this reason, Scandinavia has chosen not to implement folate fortification. In Europe, there are no legal obligations in food fortification. However, in 2014, the European Food Safety Authority^[11]^ urged women of childbearing age to fortify their diets with folates.

A safe complement to fortification with the potential to make a difference in folate status among vulnerable groups is fermentation with strains selected to produce folate during the fermentation process and/or in the human gastrointestinal tract^[12-15]^. A high folate biosynthesis capacity was found in certain species and strains of the genus *Bifidobacterium* and some yeasts. Therefore, fortification programs utilizing folate-producing microorganisms require selecting the strains most suitable for this application. Hence, the potential of folate from bacterial origin will be discussed in this review.

## HISTORY OF FOLATE INVOLVEMENT IN HOST HEALTH

Folate was first identified in the 1930s as a substance, named the “Willis factor” by Lucy Willis, that helped prevent anemia during pregnancy^[16]^. Folate was first described in 1941, when folic acid received its name after being isolated from spinach (*folium* means leaf in Latin) by Mitchell *et al*., and showed its activity as a growth factor for *Streptococcus lactis R* (now *Lactococcus lactis*)^[17]^. In the 1960s-1970s, Brown *et al*. first identified the folate pathway, the intermediates involved, and the enzymes catalyzing all reactions^[18-21]^.

## CHEMICAL STRUCTURE AND BIOSYNTHETIC PATHWAY

**Figure 1:**
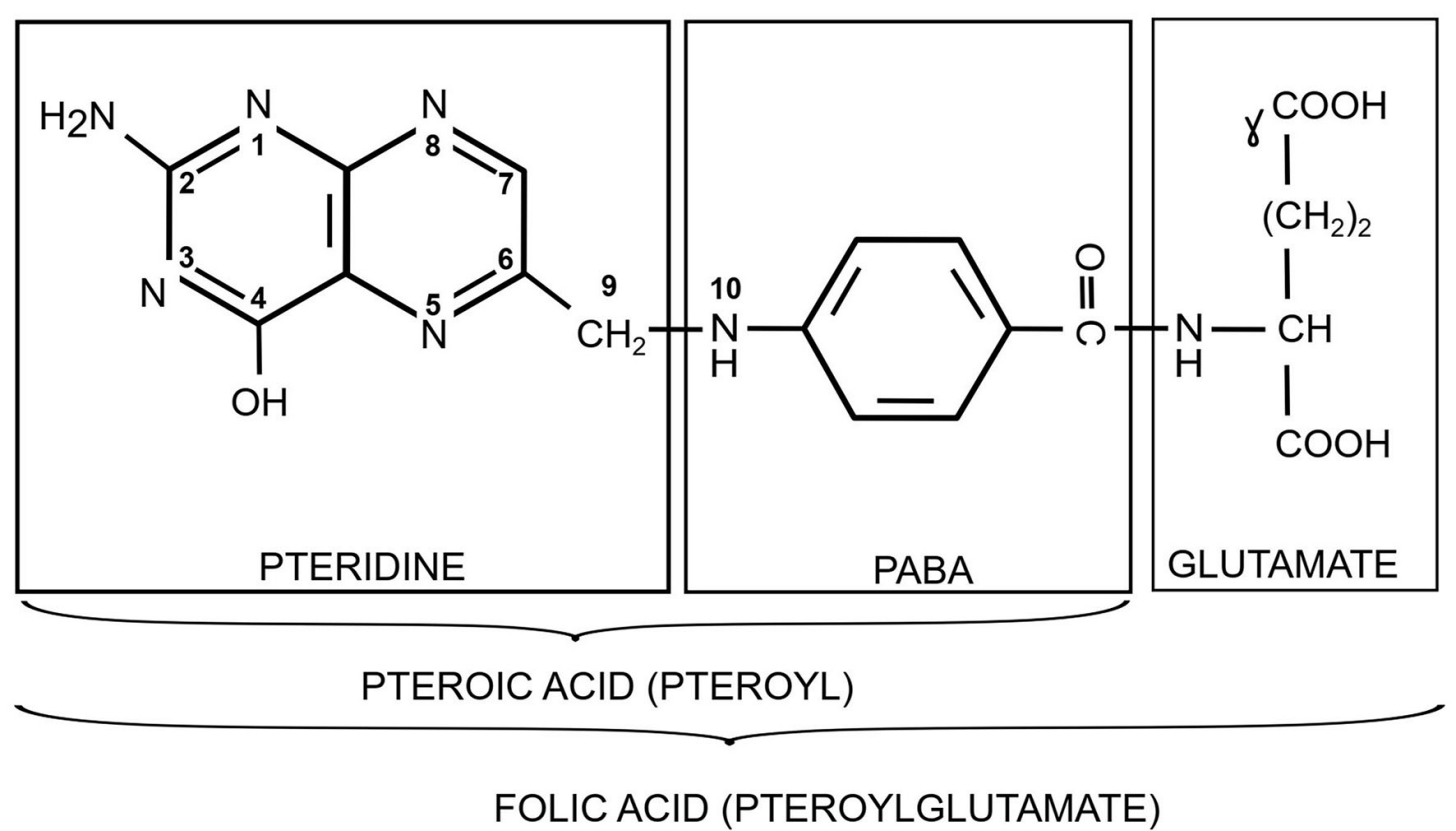
The chemical structure of folic acid (mono-glutamate derivate). PABA: Para-aminobenzoic acid.

**Figure 2:**
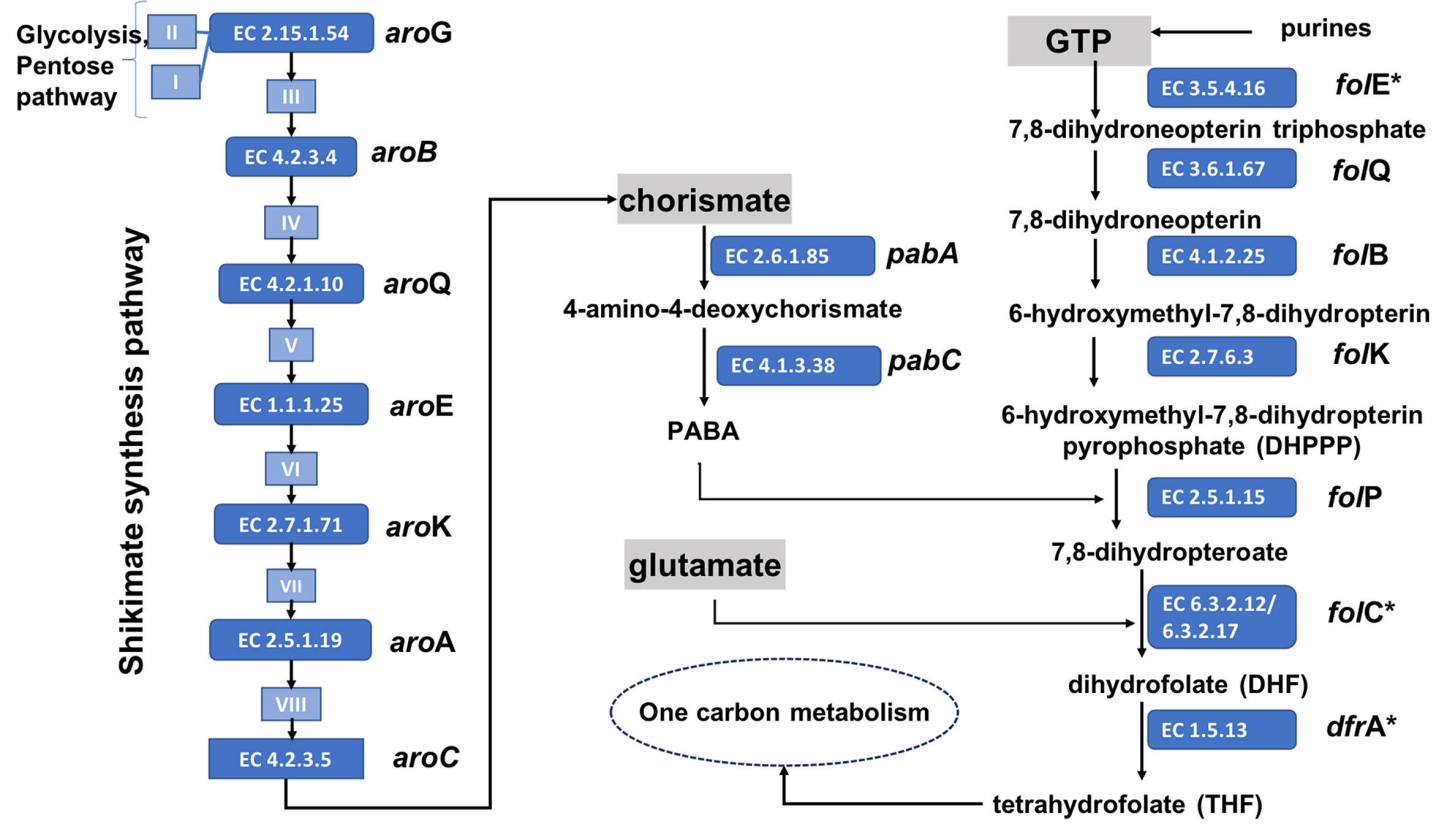
Shikimate and folate biosynthesis pathway and relevant enzymes and genes involved. Metabolites: I: D-erythrose 4-phosphate; II: phosphoenolpyruvate; III: 7-phosphate-2-dehydro-3-deoxy-D-arabinoheptonate; IV: 3-dehydroquinate; V: 3-dehydroshikimate; VI: shikimate; VII: shikimate 3-phosphate; VIII: 5-O-(1-carboxyvinyl)-3-phosphoshikimate. Enzymes: EC 2.15.1.54: 3-deoxy-7-phosphoheptulonate synthase; EC 4.2.3.4: 3-dehydroquinate synthase; EC 4.2.1.10: 3-dehydroquinate dehydratase I; EC 1.1.1.25: shikimate dehydrogenase; EC 2.7.1.71: shikimate kinase; EC 2.5.1.19: 3-phosphoshikimate 1-carboxyvinyltransferase; EC 4.2.3.5: chorismate synthase; EC 2.6.1.85: aminodeoxychorismate synthase; EC 4.1.3.38: aminodeoxychorismate lyase; EC 3.5.4.16: guanosine triphosphate (GTP) cyclohydrolase; EC 3.6.1.67: dihydroneopterin triphosphate diphosphatase; EC 4.1.2.25: dihydroneopterin aldolase; EC 2.7.6.3: 2-amino-4-hydroxy-6-hydroxymethyldihydropteridine diphosphokinase; EC 2.5.1.15: dihydropteroate synthase; EC 6.3.2.12: dihydrofolate synthase; EC 6.3.2.17: tetrahydrofolate synthase; EC 1.5.1.3: dihydrofolate reductase. In circles, genes are involved in folate biosynthesis. ^*^Enzymes common to mammals.

Folate belongs to the group of pteridines and is also known as vitamin B_9,_ vitamin M, folacin, pteroyl-L-glutamic acid, and pteroyl-glutamate. Its structure comprises three subunits: (i) a pterin moiety originating from 6-hydroxymethyl-7,8-dihydro pterin pyrophosphate (DHPPP) and (ii) a *para*-aminobenzoic acid (*p*ABA) unit linked via a methylene bridge to form the pteroic acid, which is joined by a peptide linkage to (iii) glutamic acid. The acyl group derived from the pteroic acid is a pteroyl group. Folic acid is also called pteroylglutamate (pteroyl-L-glutamic acid) [Figure 1]. Shikimate and folate biosynthesis pathways and relevant enzymes and genes involved are described in Figure 2.

While a group of bacteria possess all the enzymes to produce folate, mammals lack most of these enzymes, which explains their dependence on exogenous sources of the vitamin [Figure 2]. This finding has been supported by analysis with the software BRENDA (Braunschweiger Enzymdatenbank) (https://www.brenda-enzymes.org/), which showed that mammals possess only guanosine triphosphate (GTP) cyclohydrolase (3.5.4.16), tetrahydrofolate synthase (6.3.2.17), and dihydrofolate reductase (1.5.1.3) [Figure 2 and Supplementary Table 1].

Folic acid is a synthetic folate analog with a fully oxidized pteridine ring. Natural folates exist in their dihydro- (as in DHF) and tetrahydro- (as in THF) folate forms [Supplementary Figure 1]. Natural folates are found in foods and are all conjugated to a polyglutamyl chain containing different numbers of glutamic acids [Supplementary Figure 2] depending on the type of food; green vegetables and yeasts usually contain the eptaglutamic folate form, whereas meat contains the pentaglutamic form^[22]^.

The biological role of folates in metabolism is to donate one-carbon units in various biosynthetic pathways within cells, such as the biosynthesis of purine and pyrimidine, the metabolic transformation of glycine into serine, and the formation of glutamate from a histidine metabolite. Furthermore, folate plays an important role in homocysteine methylation, converting it into methionine by the action of the 5-methyl-THF (5-MTHF) cofactor. Owing to this reaction, the potentially cytotoxic effect of excess homocysteine levels is alleviated, and methionine is produced^[23]^. The latter is then activated to S-adenosyl methionine, the most important methyl group donor in cell metabolism^[24]^.

Dietary folates mainly exist as 5-methyl- and 10-formyl-THF in polyglutamate forms that cannot cross cell membranes^[25]^. The latter must be enzymatically hydrolyzed by folylpolyglutamate conjugase to the monoglutamyl form to be absorbed. This process occurs in the small intestine, duodenum (main absorption), distal jejunum (small absorption), distal ileum (virtually no absorption), and to a lesser extent, also in the colon. In the brush border of mucosal cells, the polyglutamyl chain is removed by the enzyme pteroyl-gamma-glutamyl carboxypeptidase (GCPII) associated with the mucous membranes of the cells and folate monoglutamate is subsequently absorbed^[26]^. The transport of folate monoglutamate inside human enterocytes is led by a proton-coupled folate transporter responsible for the intestinal uptake of folate^[26]^. In contrast to folate from foods or gut microbiota, synthetic folic acid, once in the enterocyte cytoplasm, must be reduced to THF in a two-step process led by the enzyme dihydrofolate reductase (DHFR). First, folate is reduced to dihydrofolate (DHF), which is further reduced to THF using NADPH as an electron donor^[27]^.

After absorption, folate is transported to the liver, which is estimated to store 50% of the folate in the body. The folate pool in the liver is partly secreted (intact or as inactive metabolites) into the bile^[28,29]^. Between 10%-20% of the absorbed folates are estimated to be retained in the liver^[30]^. The remaining absorbed folates are transported to other tissues via systemic circulation. In plasma, folates are partly protein-bound, mainly to albumin (50% of bound folates) but also to soluble folate-binding proteins^[31]^.

5-methyl-THF is the form in which folate moves out of the enterocyte cells to different tissues; it enters the bloodstream through the solute carrier family 19 (folate transporter). Once in the bloodstream, the delivery of 5-methyl-THF to the interior of cells is mediated by reduced folate carriers or folate receptors and polyglutamated by folylpolyglutamate synthase^[32]^. The latter converts intracellular folates into folate polyglutamates, which are poor substrates for folate transport systems and hence restricted to intracellular movement^[33]^, and passive diffusion over intact membranes is negligible due to the negatively charged carboxyl groups on each glutamate unit^[34]^. Folate polyglutamates are also better substrates for enzymes involved in one-carbon transfer^[35]^.

In cells, 5-methyl-THF can be recycled to THF in a reaction forming methionine. The step is catalyzed by methionine synthase/methionine synthase reductase (MTR/MTRR), which in humans also depends on vitamin B_12_ (cobalamin); 5-methyl-THF donates its methyl group to B_12_, which transfers it further to homocysteine resulting in methionine.

Mammals and other higher eukaryotes express only a B_12_-dependent form of MTR, whereas plants, algae, and fungi express a cobalamin-independent MTR (e.g., yeasts do not need B_12_). Other microorganisms express both the B_12_-dependent and B_12_-independent forms of MTR. Since B_12_ deficiency is also common in certain human groups, a gut colonizing strain able to synthesize both folate and B_12_, as found in some bifidobacteria, may have positive health effects.

**Figure 3:**
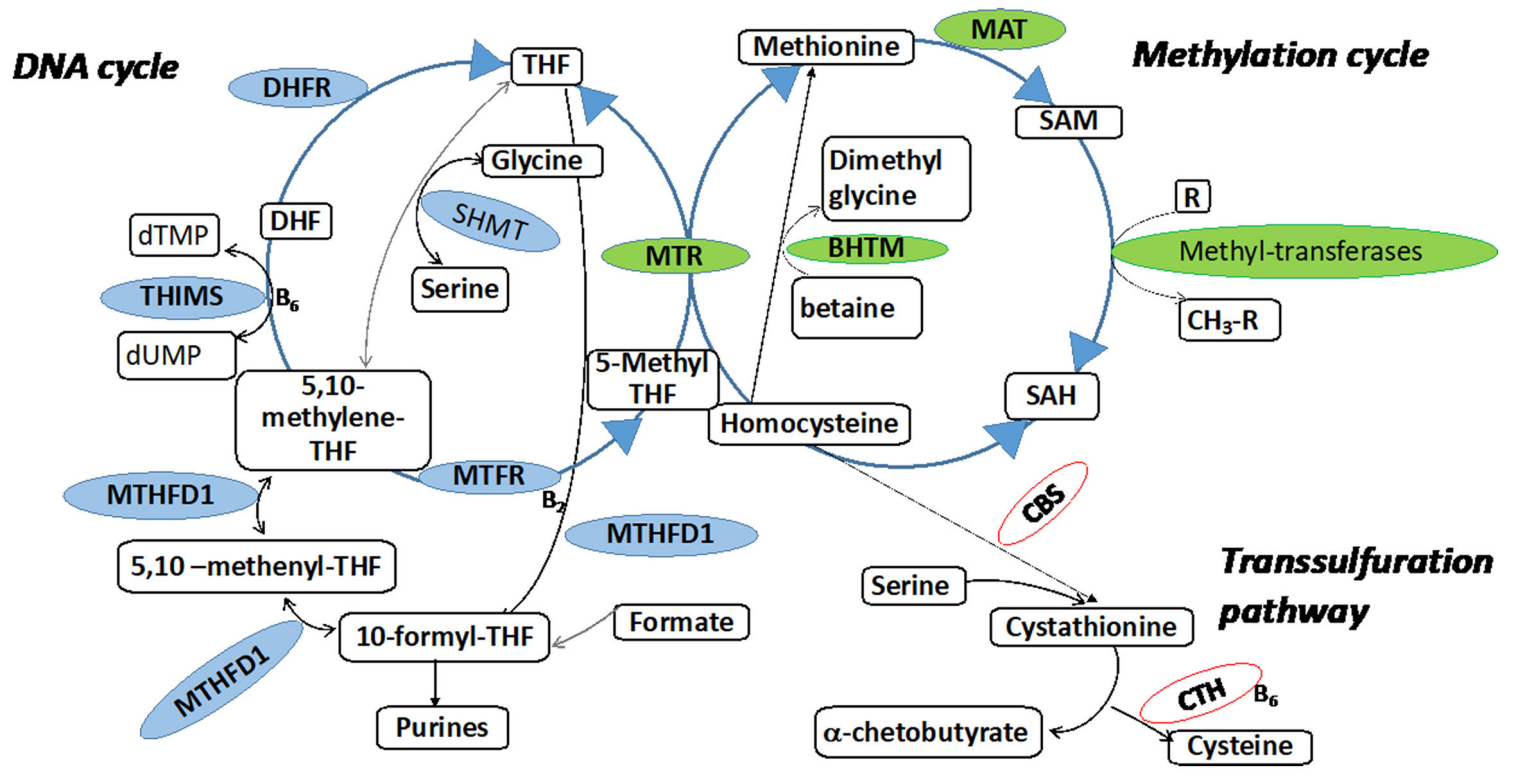
Folate-mediated one-carbon metabolism. There are two interrelated cycles in folate metabolism that compete for folate cofactors, namely DNA biosynthesis (represented by blue) and methylation (represented by green). In addition, the trans-sulfuration pathway (depicted in pink) breaks down homocysteine. 5,10-methenylTHF: 5,10-methenyltetrahydrofolate; 5,10-methyleneTHF: 5,10-methylenetetrahydrofolate; 5-methylTHF: 5-methyltetrahydrofolate; 10-formylTHF: 10-formyltetrahydrofolate; AHCY: *S*-adenosylhomocysteine hydrolase; B_2_: riboflavin; B_6_: vitamin B_6_ (pyridoxine); B_12_: vitamin B_12_; BHMT: betaine-homocysteine methyltransferase; CBS: cystathionine β-synthase; CTH: cystathionase, DHF: dihydrofolate; DHFR: dihydrofolate reductase; MAT: methionine adenosyltransferase; MTHFD1: methylenetetrahydrofolate dehydrogenase 1/methenyltetrahydrofolate cyclohydrolase/formyltetrahydrofolate synthetase; MTHFR: methylenetetrahydrofolate reductase; MTR: methionine synthase; R: methyl acceptors, such as DNA or histones; SAH: *S*-adenosylhomocysteine; SAM: *S*-adenosylmethionine; SHMT1: serine hydroxymethyltransferase 1; THF: tetrahydrofolate; TYMS: thymidylate synthetase.

The THF formed in the MTR reaction will enter the so-called DNA cycle in which it may be converted to 10-formyl-THF or 5,10-methyleneTHF (a substrate of 5,10-methyleneTHF reductase; MTHFR) and used in pyrimidine and purine synthesis, and thus DNA and RNA production. The folate form 5,10-methyleneTHF will be recycled back to 5-methyl-THF through an amino acid synthesis reaction by accepting a one-carbon group (-CH3) from the amino acid serine, which is subsequently converted into glycine^[36]^ [Figure 3].

Methionine can be used in the methionine cycle to produce S-adenosyl-methionine (SAM), S-adenosyl-homocysteine (SAH), and homocysteine [Figure 3]. Accumulation of homocysteine in the cell is thereby avoided, and synthesis of amino acids is maintained^[36]^. The conversion of SAM to SAH requires betaine, a product of the choline metabolism. SAM is the cellular methyl donor for DNA, RNA, protein, and phospholipids, and hence also central for epigenetic labeling.

## STABILITY AND INTERCONVERSION

Folates are chemically unstable reduced compounds and are particularly susceptible to being split apart by oxidative cleavage at the C9-N10 bond. This often results in the production of inactive pteridine and p-aminobenzoylglutamate molecules. However, folates with a substituent in the N5 or N10 position are more resistant to cleavage^[37]^.

This higher resistance could be attributed to a steric hindrance action against the oxidative compounds. It is worth noting that the stability of folates is affected by various factors such as pH, temperature, and the presence of metal ions such as copper and iron. Different forms of folate have varying ranges of pH stability. For instance, 5,10-CH=THF is stable at pH values below 2, whereas folic acid and 5-HCO-THF are more stable at pH values above 5^[38,39]^; 10-HCO-THF is highly unstable at all pH values. THF is also unstable, but its stability improves at pH values above 8; 5,10-CH2-THF is stable at pH above 9.5^[39]^, whereas 5-methyl-THF is relatively stable in the pH range 2 to 10^[40]^. All folate forms are sensitive to photodegradation and, therefore, require protection from UV light. The length of the glutamate tail does not interfere with the stability of the molecule^[35,41]^.

Food handling and preservation processes frequently involve steps with high oxygen levels and changing pH conditions. Folates in food, therefore, easily break down even before consumption. This decline can be attributed to factors such as exposure to oxygen, high temperatures, and light or leakage into the water used for cooking^[42]^. In addition, different cooking methods can result in varying levels of nutrient loss. Dang *et al.* examined the loss of folates in chickpeas and peas due to soaking, boiling, and pressure cooking and found that pressure cooking was the most effective method for preserving folates in peas^[42]^.

Soaking legumes during cooking can also reduce folate levels. Leakage-related losses were more significant in field peas than in chickpeas. McKillop *et al.* discovered that boiling spinach in water could lead to a 51% decrease in folate levels, whereas broccoli may lose up to 56% of its folate content^[43]^. In contrast, steaming has no noticeable impact on folate levels^[43]^. From a public health perspective, providing practical advice on cooking techniques may be a useful strategy to improve folate intake.

In addition to chemical breakdown, folates can undergo structural changes through molecular interconversion from one form to another, potentially affecting their bioavailability^[43,44]^; according to a study by O’Broin *et al.*^[45]^, folate stability depends mainly on the one-carbon unit in the molecular structure, where 5-HCO-THF is more stable than 5-methyl-THF, followed by 10-HCO-THF and THF. However, the authors quantified folates using a microbiological assay that detects all biologically active forms without qualitative information. Therefore, it cannot be ruled out that the higher stability of 10-HCO-THF over THF was due to the conversion of 10-HCO-THF to more stable 5-HCO-THF and/or 10-HCO-folic acid.

Folic acid has greater stability in comparison with the reduced folate forms. Although some folate oxidation resulting in folic acid formation may occur during food storage or cooking, folic acid is virtually unknown in nature^[46]^. This is because THF or H_2_-folate would rarely lose their hydrogen and form folic acid while remaining uncleaved^[47]^.

## ANALYSIS OF FOLATE

The methods of analyzing folates have been thoroughly reviewed elsewhere^[48,49]^, and we will only give a brief overview to emphasize some critical points [Supplementary Material]. The main steps when assessing folates in biomaterials such as microbial biomass, foods, or blood serum are: (i) extraction from the biological matrix; (ii) deconjugation of the polyglutamate tail resulting in mono-glutamate-folates; and (iii) detection of the resulting released folates. Depending on the method used, it will yield either a quantification of total folates, i.e., all bioactive derivatives counted as one, or quantitative information on each folate derivative. If total bioactive folate content is sufficient, this commonly means using a microbiological assay (MA)^[49]^. The basis for the MA is to select a microorganism that requires an external supply of folate and is unable to synthesize the folate by itself. Assuming all other necessary components are available in the medium, the growth will depend on the quantity of folate present in the added sample. By comparing the growth responses between unknown samples and folate standards, folate can be quantified. The MA is very sensitive (sub-nanogram levels) and does not require advanced analytical instruments. However, it is time-consuming and strictly quantitative and will therefore not give information on the relative concentration of different folate forms. Choosing the right species and strain of microorganisms for the MA assay is critical since it depends on equal response on growth for the different folate derivatives. *Lacticaseibacillus rhamnosus* ATCC 7469 has been shown to yield equal responses to different folate forms and has hence become standard.

If detailed information is needed, high-performance liquid chromatographic (HPLC) tends to be preferred^[50]^. By using suitable columns and methods, HPLC enables the separation and detection of individual different folate forms (typically detected by UV absorbance, fluorescence, or mass spectrometry) but requires expensive equipment and expertise [Supplementary Material].

## HUMAN FOLATE GENES AND EVOLUTION

Various environmental factors have influenced the evolution and expansion of modern humans, including the climate and diet.

During the Paleolithic era, hunting and gathering were the sole methods of subsistence, and human dietary adaptations were heavily impacted by food availability. Notably, groups inhabiting ice-age climates experienced recurring food shortages during winter and spring, resulting in a decreased intake of plant foods^[51]^. Therefore, dietary adaptations to new environments and food availability may have played a crucial role in human adaptation, including efficiency in folate uptake and metabolism, which is vital for human and animal health. Due to the low folate content in most food derived from animal sources, the diet itself was presumably insufficient to provide adequate amounts of folate. In this scenario, microbiota could have had an important role in compensating for a diet poor in folate and other important micronutrients. Folate-producing bifidobacteria may have played a key role in maintaining a sufficient folate status as an important group of the human gut microbiota.

## MICROBIAL BIOSYNTHESIS OF FOLATE AND GUT MICROBIOTA

The gut contains approximately 10^13^ to 10^14^ bacteria, roughly comparable to the average number of the human body’s cells^[52]^. These microorganisms and their genetic material constitute the gut microbiome. Most gut microbiota are anaerobic bacteria, a few hundred species per host^[53]^. Bacillota, Bacteroidota, and Actinobacteriota represent > 90% of the microbial diversity, followed by a smaller proportion of Pseudomonadota, Fusobacteriota, and Verrucomicrobiota^[54]^.

The exact timing when the gut microbiota reaches an adult-like intestinal microbiota (maturation) remains unclear. Gut colonization begins in early life and is affected by factors such as mode of delivery, breastfeeding, and gestational age at birth. After that, everyone develops a more mature and unique microbiota. However, even if the bacterial community of a mature gut is much more stable, it is not completely static but rather a dynamic system affected by lifestyle, genetic^[55]^, age, nutrition^[56]^, medications, and environment^[57]^.

Metagenomic studies found that COGs (Clusters of Orthologous Groups), involved in synthesizing B vitamins, including folate^[58]^, are enriched in the human distal gut microbiome. Bacteria can produce mono- and poly-glutamylated folate. The most common ones are polyglutamylated, such as THF, 5-methyl-THF, and 10-formyl-THF. All these forms are easily processed and absorbed by the intestinal epithelium^[59]^ of mammalian cells^[60,61]^.

The physiological surroundings, such as digestive enzymes, gut motility, acidity levels, transporters, and proteins, impact the absorption process of vitamin B_9_ in the gut. The human gut expresses two folate uptake systems. One is the proton-coupled folate transporter (hPCFT, derived from the *SLC46A1* gene), and the other is the reduced folate carrier (hRFC, derived from the *SLC19A1* gene). The hPCFT protein operates in the proximal small intestine at pH 5.5 to 6.0. On the other hand, the hRFC works better at a more neutral pH of 7.0 to 7.4 and, therefore, plays a role in uptaking folate in the distal tract of the gut under more alkaline pH conditions^[62]^. Vitamins produced by microorganisms are primarily absorbed in the colon, while vitamins supplemented with the diet are principally absorbed in the small intestine^[63]^.

The gut microbiota of mammals plays a central role in maintaining the homeostasis of folate^[64]^. Some bacteria can synthesize vitamin B_9_, while others can consume it. In addition, microorganisms can take up folate from the environment and use it in the same reactions as humans. Furthermore, another factor to consider is the amount of folate released back into the lumen when the microbiota dies and lyses. This amount, along with the balance between producers and consumers, determines the folate homeostasis in the host.

Genome analysis revealed that most lactobacilli lack the genes for the biosynthesis of pABA and are predicted or shown to be auxotrophic for folate^[65]^. As a result, *Lacticaseibacillus rhamnosus* is traditionally used to quantify folate in, for instance, food samples by microbiological assays (relying on the dependence of *L. rhamnosus* on external folate for growth see paragraph “Analysis of folate” for an explanation of the theory behind microbiological assay). However, more recently, some strains of *Lactiplantibacillus plantarum* and *Latilactobacillus sakei* have been found capable of synthesizing folate^[66]^. Many studies have focused on folate production by bifidobacteria but have not explored their potential to raise folate content when added to food products^[67]^.

Although increased folate levels in food products are possible with careful selection of strains, these levels may remain lower than the recommended daily allowance (0.2 mg of folate)^[68]^. For example, when consuming 100 g of yogurt containing folate-producing probiotics, only the minimum required folate intake for an adult may be achieved (about 15% of the minimum recommended dose). Therefore, it is important to highlight the right food quantity and variety that have to be added to the diet to meet requirements.

Our microbiome has been put forward as a nutrition source for preventing low-folate conditions. However, this potential has been hindered by the partial characterization of folate-secreting intestinal bacterial strains. Indeed, while some studies have evaluated probiotic strains of *Bifidobacterium* spp. and *Lactobacillus* spp., as previously mentioned, little is known about the capacity of gut microbiomes to generate folate.

While all Eubacteria and Eukarya require folate, some Archaea (*Methanobacterium thermoautotrophicum*, *M. thermoautotrophicum*, and *Sulfolobus solfataricus*) do not and may have evolved alternative pathways for purine biosynthesis^[69]^. Recent data show alternative non-folate carbon group carriers in Archaea in metabolic steps where folate is required in bacteria and Eucaryota.

Engevik *et al.* showed folate biosynthesis as a major biochemical feature of the human intestinal microbiome^[70]^. Four metabolic modules in folate biosynthesis (chorismate, pABA, pterin, and folate synthesis) have been described in terms of prevalence in a reference set of 512 microbial genomes accredited to the human gastrointestinal tract. The gene distribution was studied among six phyla of the human microbiome: Actinobacteriota, Bacteroidetes, Firmicutes, Proteobacteriota, Fusobacteriota, and Verrucomicrobiota.

The authors found that 13.3% of the bacterial reference genomes contain genes involved in *de novo* synthesis of THF. Therefore, the gut microbiome can be considered a folate-generating “organ”. The remaining genomes (circa 87%) require folate or folate intermediates from other bacteria or the human diet.

Engevik’s and colleagues’ data were consistent with previous genomic studies that predicted 79% of Fusobacteria, 71% of Proteobacteria, 26% of Actinobacteria, and 15% of Firmicutes in the human intestinal microbiome can produce folate *de novo*.

In a study by Magnúsdottir *et al.*, the genomes of 256 common human gut bacteria were assessed for biosynthesis pathways for eight B vitamins (including folate) using the PubSEED platform^[71]^. It was predicted that 40%-65% of the gut microbiota could produce each of these vitamins. The authors hypothesized that the coevolution of the human gut microbiota led to the shared B-vitamin biosynthesis capability among these bacteria. Their data showed that the folate biosynthesis pathway was present in nearly all Bacteroidetes genomes and most Fusobacteriota and Proteobacteria. However, genes for folate synthesis were rare in the genomes of Actinobacteria and Firmicutes groups, mostly because of the absence of the pABA biosynthesis pathway. They speculated that the human microbiome could synthesize 37% of the daily recommended folate intake for non-pregnant, non-breastfeeding adults.

Nonetheless, establishing a clear connection between the existence of folate biosynthesis machinery and its actual *in vivo* activity level presents a challenging and currently elusive task. From our perspective, there is a substantial need for further research to determine the extent to which the gut microbiota contributes to human folate intake.

## THE PRODUCTION OF FOLATE IN BIFIDOBACTERIA

The genus *Bifidobacterium* is considered one the most important bacterial group of the gut microbiota, exerting various health-promoting effects on its host: bifidobacteria supports the development of the host immune system, promote intestinal barrier integrity, and provide protection against pathogen growth^[72,73]^. The *Bifidobacterium* genus includes several species capable of *de novo* folate biosynthesis^[58,65,74-76]^.

Folate content in a particular bacterium is not a static property unless conditions are steady, such as in a chemostat, which is never the case in real-life scenarios. Moreover, another significant factor is that the folate content can change significantly based on various factors, such as the type and degree of cellular activity, population state, growth rate, pH, temperature, and medium composition^[77-79]^. For example, folate-producing LAB strains exhibited a temperature-dependent pattern. At 42 °C, they achieved maximum folate production in just 6 h, whereas at 37 °C, it took 8 h to reach the same level. This highlights the crucial role of temperature control in optimizing folate production in LAB strains^[80]^. Controlled pH conditions favored the synthesis of folate in *Streptococcus macedonicus* CRL415: In fact, the total amount of folate produced raised ca. 2-fold when the pH was increased from 5.0 to 6.0 with respect to free-pH^[81]^. Therefore, the folate content of a bacterium is highly variable and subject to change. Another important factor contributing to the diversity is the variation in folate analysis methods used in different studies. Finally, factors contributing to biodiversity are natural and intrinsic gene variation. This is supported by research on numerous strains under identical conditions in a single laboratory^[74,82]^.

The predominant phenotype observed in certain species is linked to functional folate-synthesizing machinery. Among these species, some have consistently demonstrated a high specific folate content, measured as the weight of folate per dry weight of bacterial biomass. Notably, *B. adolescentis*, *B. bifidum*, *B. catenulatum*, and *B. pseudocatenulatum* are among the studied species with a dominant autotrophic ability for folate synthesis.

In *B. catenulatum* ATCC 27539, the highest value of folate production previously found was 93 µg per g DM (the highest in yeast is approximately 200 µg per g DM). The substantial diversity observed in yeasts and bifidobacteria can be attributed to technical and biological factors. As a point of comparison, broccoli, renowned for its folate content, typically contains about 70-80 µg of folate per 100 g of fresh weight. Considering its water content of about 87%, this translates to approximately 5.5 µg/g of dry weight (17 times less than the folate content of *B. catenulatum* ATCC 27539). It is uncommon for humans to consume large amounts of microbial biomass, except for yeast. Nonetheless, the high specific levels of bifidobacteria and yeasts can still have a notable impact on fermented foods.

*Bifidobacterium animalis* subsp. *animalis* ATCC 25527 is a species in which folate biosynthesis has been found absent, with the lowest level per biomass unit corresponding to 2 µg per g DM^[74]^.

In addition, it is also crucial that the folate-producing strains should be able to grow consistently and in high amounts under the desired conditions to produce them easily for application. Therefore, the amount of folate per unit biomass is one of the key factors considered during the screening and development of folate-producing strains, along with the ease of biomass production and yield obtained from suitable raw materials. Furthermore, folate-producing probiotics must meet several other requirements, such as fulfilling the EFSA^[11]^ criteria and actively producing folate in the gut ecosystem.

The idea of folate trophic probiotics seems feasible and should be explored further. It is conceivable that bifidobacteria adapted to the human colon have been selected for folate production because of the mutual benefits it provides.

## BIOSYNTHESIS OF FOLATE BY BIFIDOBACTERIA: FROM GENES TO PHENOTYPE

### *In silico* studies for folate production

The complete biochemical pathway of folate biosynthesis in bifidobacteria is still being investigated. By comparing identified folate biosynthesis genes present in other organisms with bifidobacterial genomes, genome information can be used to identify genes and corresponding proteins that are likely involved in folate biosynthesis^[65]^.

Several databases and web resources are available for studying microbial genomes. The US Department of Energy Joint Genome Institute (DOE JGI) established the online Integrated Microbial Genome System (IMG)^[83]^ as a genome search and annotation platform that supports distribution and genome analyses. With databases such as IMG and MicrobesOnline^[84]^, it is possible to study and compare microorganisms’ genomes. For example, these databases allow researchers to check whether a microorganism has examples of all the necessary genes to produce a vitamin such as folate. However, the mere presence of genes does not guarantee that the corresponding proteins are functional.

**Figure 4:**
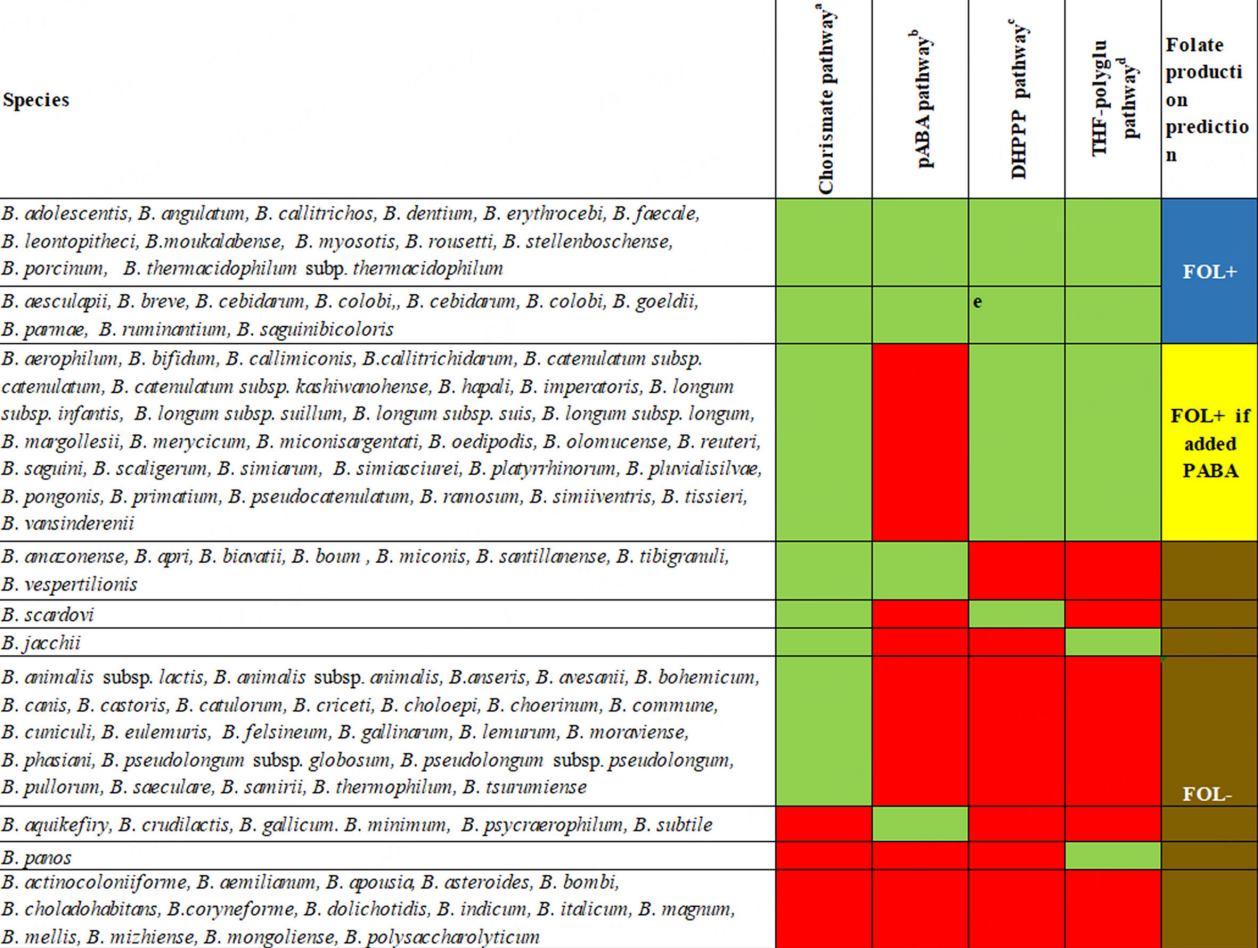
Presence of genes for the biosynthesis of chorismate, pABA, DHPPP, and THF-polyglutamate pathways and prediction of folate synthesis from the sequenced genomes of type strains of bifidobacterial species so far described. Chorismate pathway comprises aroG 2.5.1.54, aroBK 4.2.3.4, aroQ 4.2.1.10, aroE 1.1.1.25, aroBK 2.7.1.71, aroA 2.5.1.19 and aroC 4.2.3.5; ^b^pABA pathway comprise pabA 2.6.1.85 and pabC 4.1.3.38; ^c^DHPP pathway comprises folE 3.5.4.16, 3.1.3.1, 3.6.1.-, folBK 4.1.2.25, folBK 2.7.63; ^d^THF-polyglud pathway comprises folP 2.5.1.15, folC 6.3.2.12/17, dfrA 1.5.1.3. Rectangels in green: all genes of the pathway are present; rectangels in red: at least one gene of the pathway is absent. In the right you will find the folate production prediction based on th epresence of pathwas genes: in blue: predicted folate production; in yellow: pABA needed for predicted folate production; in brown, no predicted folate production; ^e^all the genes for the biosynthesis of DHPPP are present, with the exception of alkaline phosphatase (EC 3.1.3.1). The dephosphorylation of dihydroneopterin triphosphate into the monophosphate can occur through an alternative route using pyrophosphohydrolase number EC 3.6.1.-. which is present (Rossi 2011).

All known type strains of the species of bifidobacteria have been sequenced to date. At the time of writing, the databases contained genome information about the 111 currently described species of bifidobacteria (https://site.unibo.it/subcommittee-lactobacillus-bifidobacterium/en). *In silico* analysis of the folate biosynthesis genes for all 111 bifidobacterial species was performed. The species genomes were examined for the presence of genes involved in the biosynthesis of DHPPP, THF-polyglutamate, chorismate, and pABA [Figure 2]. As *B. adolescentis* is known to be capable of *de novo* folate production, the genes from *B. adolescentis* were taken as a reference for BlastP searches. Based on the identity values, strains in Figure 2 are marked as being involved in folate biosynthesis or not. The identity value of 50% is used as a threshold to define the presence or absence of a gene in a particular strain.All 111 bifidobacterial species have been found to possess the required genes to produce chorismate by yielding shikimate [Figures 2 and 4].

**Figure 5:**
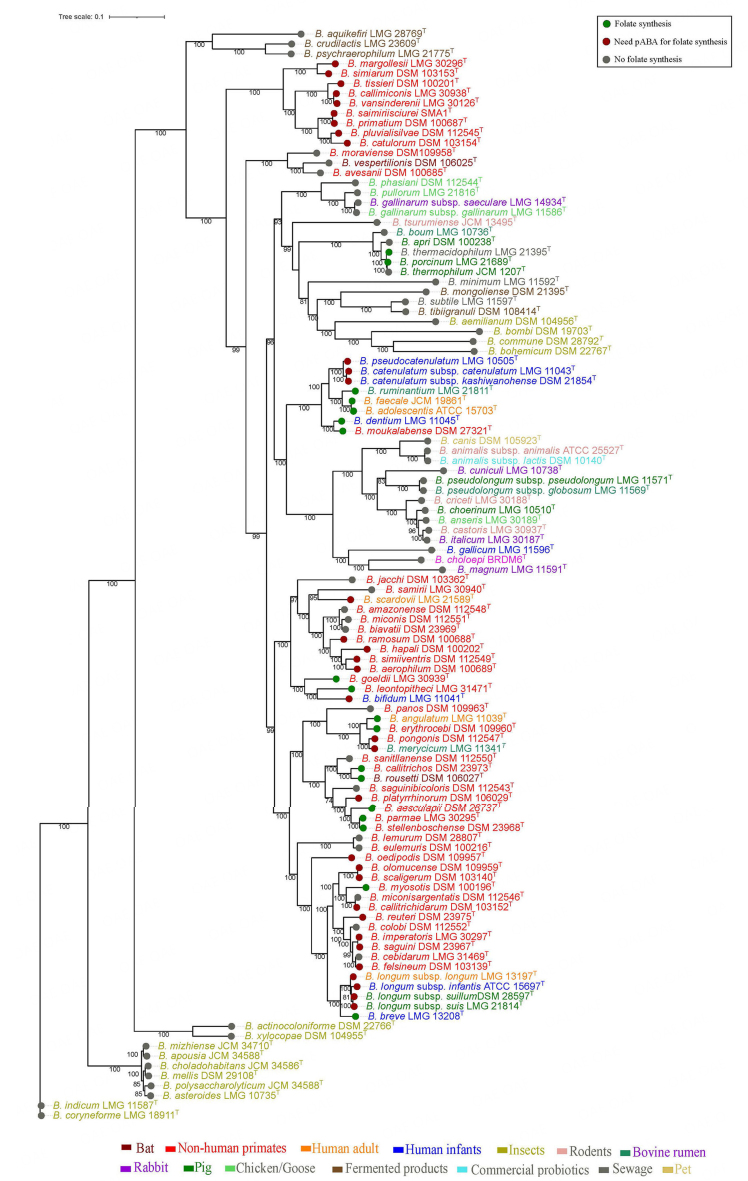
Phylogenomic tree of *Bifidobacterium* genus describing the origin and the ability of the type strains of species to produce folate.

In general, *de novo* folate biosynthesis requires the precursors of both DHPPP and pABA. Upon analyzing the genomes, it was found that most species carry the *pab*A gene, which encodes aminodeoxychorismate synthase for pABA biosynthesis. However, only *B. adolescentis*, *B. angulatum*, *B. breve* and *B. dentium* that are typically found in humans, *B. aesculapii*, *B. callithricos*, *B. callithrichidarum*, *B. goeldii*, *B. myosotis*, *B. moukalabense*, *B. parmae* and *B. stellenboschense* from non-human primates and *B. ruminantium*, *B. porcinum*, *B. rousetti* and *B. subtile* from other animals have the *pab*C gene, that encodes the 4-amino-4-deoxychorismate lyase and are considered able to complete the pABA biosynthesis process [Figure 4 and Supplementary Table 2]. The bifidobacterial phylogenetic tree of the 111 species tested showed that folate-producing species cluster in the same branches [Figure 5].

All human-derived species, except *B. gallicum*, are prototrophic or require pABA for folate production. For bifidobacterial species in non-human primates^[85-87]^, about one-third are prototrophic, one-third can produce folate in the presence of pABA, and one-third are auxotrophic for the folate; moreover, most bifidobacterial “animal species” were found to lack crucial genes, namely *fol*E and *fol*BK, which encode the enzyme 6-hydroxymethyl-7,8-dihydropterin pyrophosphokinase (HPPK) responsible for DHPPP biosynthesis, as well as the *fol*P gene encoding dihydropteroate synthase, an enzyme involved in the condensation of DHPPP and pABA^[5,88]^ [Figure 2]. Therefore, it was concluded that animal species, with few exceptions, are auxotrophic for folate, even with the presence of pABA [Figure 4]. On the other hand, most of the bifidobacterial species from human and non-human primates have the set of *fol* genes but require the supplementation of pABA^[65]^.

### *In vitro* studies for folate production

*In vitro* studies tested 76 strains belonging to different species of the genus *Bifidobacterium*, isolated from both humans and animals, and found 17 strains to be folate-producing^[62,63]^. All these strains were of human origin, thus suggesting a higher incidence of folate-producing bifidobacteria in humans as compared to other animals, a finding confirmed by D’Aimmo *et al.*^[74]^. These studies also confirmed folate production variation between strains within, as well as between species.

In another *in vitro* folate production study, it has been shown that in the absence of pABA, folate production was suppressed or reduced for many strains. Moreover, increasing the concentration of pABA led to great variability in folate production^[82]^. Only for strains of *B. adolescentis* and *B. pseudocatenulatum*, the pABA concentration did not affect the final folate content. In the same study, any relation between carbon source and folate production was excluded, as well as the effect of pH. Furthermore, this vitamin’s concentration was higher than the cell’s requirements in some cases. For this reason, it is possible to hypothesize that biosynthesis is not regulated in some strains^[82]^. Regarding *Bifidobacterium* folate composition, the predominant form in most strains tested by D’Aimmo *et al.* is 5CH_3_H_4_ folate, followed by H_4_-folate^[58,74]^. The HCO-H_4_ folate was not detected in *Bifidobacterium* spp.

Comparison of phenotypic and *in silico* data on folate production in bifidobacteria showed some inconsistencies due to the quantification method used. Some studies^[58,74,89]^ confirmed the data obtained *in silico*, i.e., strains of human origin (in *in silico* study, all human bifidobacterial species contain folate genes) can grow without folate supplementation [Table 1]. However, if a different quantification method was used, as in the survey of Pompei *et al.*^[82]^, different results were obtained, which did not completely support the *in silico* data: indeed, these authors showed that 41 out of 57 tested bifidobacterial strains of human origin did not produce folate in the folate-deficient medium (see for example results obtained for *B. adolescentis* ATCC 15703 and *B. longum* subsp. *longum* ATCC 1570). By using HPLC folate detection assay, D’Aimmo *et al.* and Sugahara *et al.* found that all tested bifidobacteria of human origin could grow and produce folate in folate-deficient media, thus suggesting that differently than in animals, all human resident bifidobacteria can produce folate^[58,74,89]^. Therefore, the importance of appropriate methods for evaluating folate production by bifidobacterial strains has to be underlined.

**Table 1:** Phenotypic production of folate by bifidobacterial species

**Species**	**Folate-producing species**	**Non-folate-producing species**
Humans	*B. adolescentis*, *B. bifidum*, *B. breve*, *B. catenulatum*, *B. dentium*, *B. longum* subsp*. infantis*, *B. longum* subsp. *longum*, *B. pseudocatenulatum*	
Non human primates	B. catulorum^*^, B. hapali^*^, B. myosotis^*^, B. tissieri^*^	B. eulemuris^*^, B. jacchi^*^, B. lemurum^*^
Other animals		*B. asteroides*, *B. animalis subsp. animalis*, *B. animalis* subsp. *lactis*, *coryneforme*, *B. indicum*, *B. pseudolongum* subsp*. globosum*

^*^Data not shown, personal communication; data from Pompei *et al.*^[82]^; D’Aimmo *et al.*^[58,74]^; Sugahara *et al.*^[89]^.

Another outcome in the phenotypic study of folate production is the high variability in the amount of folate produced between different strains^[58,74,82,89]^. Screening for folate production of the bifidobacteria isolates from human adults and infants (1-6 months old) showed that strains derived from adults were the higher producer of total folate (range from 580 to 935 μg/g dry matter) with the predominance of 5-methyl-H_4_ folate and a low amount of H_4_ folate^[74]^. In infants, the opposite results have been described with strains typical of infant habitat producing low amounts of total folate (range from 35 to 200 μg/g dry matter) and an inverted ratio of 5-methyl-H_4_/H_4_ folate concerning adults. In agreement with the previously described idea of coevolution of host-gut microbiome^[90]^, it can be hypothesized that bifidobacteria present in the adult gut were able to produce a high amount of folate, differently from strains derived from infants. These findings could correlate with the diet and the folate requirement of the host: in infants, milk feeding is able per se to fulfill the folate needs of the individuals, whereas, in adults, a more complex diet is sometimes unable to cover all the folate needs. The relevance of the different ratios of 5-CH_3_-H_4_/H_4_ folate production in adults and infants has been studied only in a few strains. Further studies are requested to have more evidence and provide an ecological explanation.

### *In vivo* studies for folate production

*In vivo* studies of folate production by bifidobacteria are very scarce. Pompei *et al.*, for instance, evaluated *in vivo* folate production in Wistar rats^[82,91]^. Folate-overproducing bifidobacteria *B. adolescentis* MB 227, *B. adolescentis* MB 239, and *B. pseudocatenulatum* MB 116 were administered, with or without prebiotics, to rats with induced folate deficiency. A higher increase of serum folate (16.4 ± 3.7 nmol/L) was found in the group of animals receiving probiotic and prebiotic (fructo-oligosaccharide) compared to the group receiving only probiotics (9.1 ± 0.3 nmol/L). Nevertheless, the rats fed probiotics showed a greater folate increase than the control (4.8 ± 0.5 nmol/L) and than the prebiotic-only group (5.3 ± 1.4 nmol/L). This suggests that ingesting folate-producing bifidobacteria may increase folate absorption into the bloodstream. However, the impact on folate levels in humans may be somewhat less significant, as rats engage in coprophagia, which results in an internal increase of folate for themselves.

To the best of our knowledge, only one study in humans was carried out and showed inconsistent data: Strozzi and Mogna (2007) found an increase of folate in fecal samples^[92]^. However, the strains added to the diet belonged to *B. animalis* subsp. *lactis*, a species that, *in vitro* and *in silico*, does not show the ability to produce folate. It is not to be excluded that the folate production could be derived from interactions of *B. animalis* subsp. *lactis* with other microorganisms in the gut microbiota, which could potentially provide the necessary intermediates for folate production. Therefore, new studies will be requested to allow more evidence about the *in vivo* production of folate by bifidobacteria and their benefit for the host.

## BIOTECHNOLOGY APPROACH FOR FOLATE FORTIFICATION

Due to limited monitoring, the extent of folate deficiency worldwide remains poorly understood. However, it is evident that vulnerable groups, such as pregnant women, the elderly, and those with limited intake of legumes, leafy vegetables, and fruits, are at a higher risk of being deficient. This highlights the need to explore the use of microorganisms to increase folate levels in food and enhance its absorption in the gut. Many countries have already implemented mandatory fortification of certain food products with folic acid.

Several studies raise reservations regarding the safety of high intake of chemically synthesized folic acid in foods, whereas naturally produced folates are not known to cause such health concerns^[67,68]^. Natural folates are typically in a form that the body can readily use and, as they occur in foods, are generally considered safe without the risk of excessive intake. In the case of synthetic folic acid, on the other hand, the liver needs to convert it into its active form (tetrahydrofolate) before it can be utilized. Concerns have been raised that overdosing on synthetic folic acid is linked with the development and progression of certain cancer forms^[93,94]^, masking of vitamin B_12_ deficiency, and thus the risk of developing neuropathy^[59]^ and reducing the effectiveness of some medications.

However, research initiatives to evaluate the potential of natural folate production by microbial fermentation could offer solutions for folate supplementation without the potential risk of high doses of chemically synthesized folic acid.

An interesting approach is using folate-producing strains to increase folate levels in fermented foods; different starter and probiotic cultures have been shown to produce folate^[95,96]^. Microbial production can be regarded as a sustainable technology since it is based on the fermentation of renewable resources, such as crops, fruits, vegetables, and their processing sidestreams. Optimal mixes of folate compounds could be produced economically and favorably through the biofortification of foods with starter cultures controlled fermentations. This would allow the production of food items with an improved vitamin content at lower cost, and therefore, specifically useful in developing countries^[97]^.

For example, the folate content in the traditional Tanzanian cereal-based food/beverage, togwa, was increased 20-fold (from 3.4 μg/100 g to 69 μg/100 g of dry matter) using selected strains in model fermentations^[15]^. In another example, everyday wheat bread was fortified with folate by employing a specific yeast strain and bioprocessing. This resulted in four times higher folate levels than bread made with a commercial baker’s yeast strain (from 35 μg/100 g to 137 μg/100 g of dry matter)^[12]^. Kefir-strains of yeast are also promising in increasing folate content^[14]^. Additionally, oat bran can be fermented to improve its folate content, as shown by Korhola *et al.*^[98]^.

Other studies focussed on a traditional African cereal-based fermented food called ben-saalga, a pearl-millet-based fermented porridge consumed in Burkina Faso^[99]^. The microbiota in ben-saalga has the genetic potential for folate production^[100]^. The total amount of folate in the soaked and fermented grains was significantly higher (circa 30% more) than in the original pearl-millet grains.

Some studies investigated the ability of bifidobacteria to increase folate content directly in fermented milk, indicating their potential use in obtaining fermented dairy products^[77,101,102]^. Thirty-two *Bifidobacterium* isolates (*B. lactis* spp., *B. animalis* spp., *B. infantis* spp., *B. breve* spp.) were examined for folate production while fermenting skim milk. Of these strains, *Bifidobacterium breve* 5181 resulted in the most significant increase in folate levels. The *B. animalis* (CSCC 1941) and *B. lactis* (CSCC 5123, CSCC 5127, Lafti B94) isolates roughly doubled the folate concentration in the skim milk^[101]^. However, in a different study, *Bifidobacterium bifidum* was found to deplete 5-methylTHF in milk by approximately 0.15 µg/100 g after 12 h of fermentation^[103]^. However, some strains reduce 5-methyl-THF in milk, suggesting the importance of strain selection^[103]^. This microbial-based approach in food fermentations is an attractive alternative to synthetic folic acid fortification, especially considering that the active form of folate in microorganisms (THF) is identical to that in humans.

To fulfill human vitamin B_9_ needs, utilizing a co-culture of different bacterial strains and yeasts, alongside the selection of strains with high folate production capabilities, could be more effective than using single cultures. For example, in reconstituted skim milk, a co-culture of two *B. animalis* subsp*. lactis* and *L. acidophilus* strains produced at least 30% more folate than the single cultures^[101]^.

A few studies have also registered the use of metabolically engineered industrial microorganisms, such as *Escherichia coli*, *Bacillus subtilis*, LAB (Lactic Acid Bacteria), and the fungus *Ashbya gossypii* capable of overproducing folate. Consumption of food fermented with overproducing LAB strains improved the folate status in deficient rats^[64]^. The use of genetically modified microorganisms may be limited by ethical and regulatory considerations. However, such studies are important to demonstrate the possibility of this biotechnological approach for industrial folate production^[104]^.

In the near future, the nutritional analysis may combine the most suitable associations of probiotics (microbiome data) and precise dietary information to target people’s needs so that folate deficiency can be prevented (“personalized medicine”).

As research for folate-producing starter strains in food fermentation is still nascent, there is a substantial potential to enhance the natural folate content in fermented foods. It has been shown that growth rate, physiological state, and medium composition strongly affect the specific folate content in yeasts. A manyfold higher folate content was obtained in fermenting fast-growing yeasts compared with respiring slow-growing yeast^[105]^. Similar variations have also been observed in bifidobacteria^[74]^. It is of high importance to not only optimize strain but also the process.

Therefore, microbial metabolic engineering, which involves the modification of metabolic pathways through genetic manipulation, represents a potent tool for enhancing the production of valuable compounds in microorganisms beyond their inherent capabilities. These customized microbial cell factories, referred to as engineered microbes, have been effectively generated to overproduce various vitamins like B_2_, B_12_, and C^[106-108]^. However, it is worth noting that folates continue to be synthesized chemically, as previously mentioned. Nevertheless, numerous metabolic engineering strategies have emerged in both prokaryotic and eukaryotic microorganisms, paving the way for a more sustainable approach to vitamin B_9_ production in the near future.

Ultimately, to achieve biotechnological folate production and biofortification of fermented foods, two essential factors must be considered: (i) robust biomass production of microorganisms and (ii) an appropriate technical process. The microorganism used should have the ability to produce folate efficiently in a biological reactor, fermented foods, or the gut microbiota and must possess suitable properties to complete that. The latter approach is particularly attractive since folate probiotics can significantly enhance folate uptake by the host animal, as discussed above. The technological process must promote the production and accumulation of folate in the chosen bioreactor or food product.

## CONCLUSIONS

The global prevalence of folate deficiency varies largely between countries and surveys. Meta-analysis of relevant studies suggests > 20% in low-income countries and < 5% in high-income countries. Deficiency increases the risk for various negative health consequences, such as birth defects and anemia.

The growing awareness of the complexity and importance of the human gut microbiota for health and disease motivates further studies targeting specific questions. This review addressed whether bifidobacteria may contribute to a healthy folate status. The present review shows that fundamental research within this field will lead to crucial insights such as recognizing a “good folate gut microbiota” *vs*. a “not so good”.

There are many open questions to be asked in the coming research. Many of those require a metagenomic sequencing of the gut microbiota composition and an assessment of folate status in animals and humans. How does it correlate? We would like to propose the following questions: (i) does a folate-rich diet select a different gut microbiota compared to a folate-poor diet? If yes, (ii) can a gut microbiota with a large fraction of high folate-producing bacteria compensate for a folate-poor diet to some extent? (iii) Can we significantly improve the human folate status in those needed by selected bifidobacteria as probiotics and/or in fermented foods? If yes, (iv) would it depend on the individual gut microbiota present? In other words, could we learn how to recognize a gut microbiota likely to respond well to certain “folate probiotics”?

The bifidobacteria folate biosynthesis machinery and related genetics and physiological and bioprocessing studies on folate production were reviewed. The differences between species and strains are large, as is the impact of cultivation conditions on specific folate content. Advancing comprehension in this field will contribute to the formulation of bio-based strategies aimed at enhancing folate levels in disadvantaged population groups. This motivates further R&D to develop naturally high-folate fermented foods and probiotics as alternatives to traditional fortification with synthetic folic acid.

## DECLARATIONS

### Copyright

© The Author(s) 2023.
