# Supplementary material for: Folate-producing bifidobacteria: metabolism, genetics, and relevance
Source: Microbiome Res Rep. 2023 Dec 12;3(1):11. doi: 10.20517/mrr.2023.59 (PMC10917623; doi:10.20517/mrr.2023.59)
Supplement: Supplementary file 2 [file mrr-3-1-11-SupplementaryMaterials.pdf]

Supplementary Figures

Folate-producing bifidobacteria: metabolism, genetics, and relevance

Maria Rosaria D'Aimmo[1], Maria Satti[2], Donatella Scarafile[2], Monica Modesto[2], Stefano Pascarelli[3], Simone Andrea Biagini[4], Donata Luiselli[5], Paola Mattarelli[2], Thomas Andlid[6]

[1]BioGaia AB, Bioventure Hub, HE424, c/o AstraZeneca AB, R&D, Mölndal 43150, Sweden.
[2]Department of Agricultural and Food Sciences, University of Bologna, Bologna 40127, Italy.
[3]Protein Engineering and Evolution Unit, Okinawa Institute of Science, Technology Graduate University, Okinawa 40-0193, Japan.
[4]Institut de Biologia Evolutiva (UPF-CSIC), Departament de Medicina i Ciències de la Vida, Universitat Pompeu Fabra, Parc de Recerca Biomèdica de Barcelona, Barcelona 08003, Spain.
[5]Department for the Cultural Heritage (DBC), University of Bologna, Ravenna 48121, Italy.
[6]Andlid Bio Solutions AB, Gothenburg 41871, Sweden.

Correspondence to: Dr. Monica Modesto, Prof. Paola Mattarelli, Department of Agricultural and Food Sciences, University of Bologna, Viale Fanin 44, Bologna 40127, Italy. E-mail: monica.modesto@unibo.it; paola.mattarelli@unibo.it

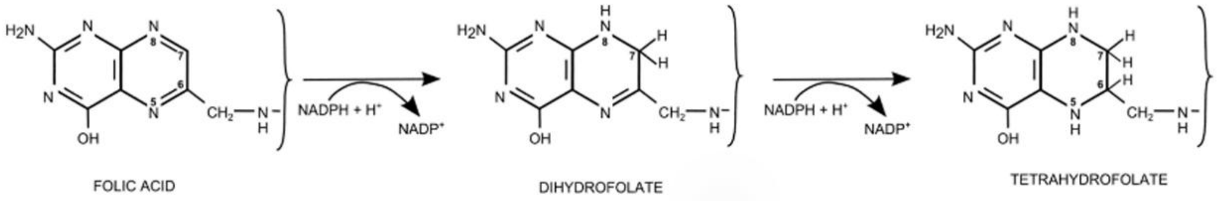

Supplementary Figure 1. The structure of folic acid, dihydrofolate, and tetrahydrofolate.

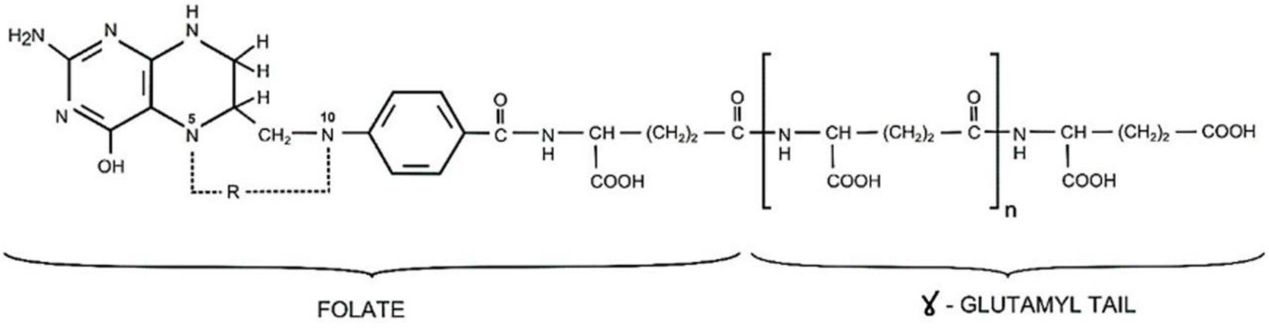

Folates derivatives	R substitute at N^5	R substitute at N^{10}	Bridge (N^5 - N^{10})
THF	-H	-H	
5-CH_3-THF	-CH_3	-H	
5-NHCH-THF	-CH=NH	-H	
5-HCO-THF	-HCO	-H	
10-HCO-THF	-H	-HCO	
5,10-CH_2-THF			-CH_2-
5,10-CH^+=THF			-CH^+=

Supplementary Figure 2. The structure of folate and its derivatives. Folates commonly have a γ-polyglutamyl tail linked to the first glutamate ($n \leq 8$ residues). Different one-carbon units (R substitutes) can be linked to the N^5 and/or N^{10} positions.
