# Supplementary material for: Folate-producing bifidobacteria: metabolism, genetics, and relevance
Source: Microbiome Res Rep. 2023 Dec 12;3(1):11. doi: 10.20517/mrr.2023.59 (PMC10917623; doi:10.20517/mrr.2023.59)
Supplement: Supplementary file 3 [file mrr-3-1-11-SupplementaryMaterials1.pdf]

Supplementary Material

Analysis of bacterial folates

The main steps to assess folates in biomaterials such as microbial biomass, foods or blood serum are described below.

Extraction

The sample preparation is often given the least attention but will have a significant impact on folate availability for the steps to follow; deconjugation and detection. For a complex and/or rigid sample such as many foods, grinding and homogenization (often of a lyophilized sample) in a suitable buffer system followed by heating and centrifugation is usually necessary[1]. Heating during the extraction procedure causes thermal denaturation of folate-binding proteins and enzymes that may catalyze folate degradation or interconversion and also precipitate structural proteins (Gregory, 1989; Keagy, 1985), which may aid the folate release. Natural folates are however unstable and susceptible to thermal and oxidative degradation (see section X), and need to be stabilized during the whole procedure (Strandler *et al.*, 2015). Samples should be protected from UV light, oxygen, and metal ions, such as copper and iron, that can promote free radical oxidation reactions. All analytical steps should be optimized toward shorter incubation times and lower temperatures everywhere it does not deteriorate the yield of folate (Strandler *et al.*, 2015).

For analysis of the folates within microorganisms such as bifidobacteria and yeasts, it has been found that lyophilized microbial biomass can be extracted effectively without need for prior mechanical destruction (Hjortmo *et al.* 2005 Trends in Food Science & Technology 16 (2005) 311-316; D'Aimmo *et al.*, 2012).

The combination of two antioxidants; ascorbic acid and a thiol, has been found most efficient to protect folates from degradation (Wilson and Horne, 1984). The combination of ascorbic acid with 2-mercaptoethanol (Eitenmiller *et al.*, 2008; Wilson and Horne, 1984) has also been used. However, 2-mercaptoethanol is less effective and more toxic than other thiols, such as 2,3-dimercaptopropanol, 1,4-dithiothreitol, or 2-thiobarbituric acid (Patring *et al.*, 2005). Using an HPLC method developed for yeast, 2-mercaptoethanol was successfully replaced by more efficient and the less toxic thiols 2,3-di-mercaptopropanol for bifidobacteria[2] and 1,4-

dithiothreitol[3].

Deconjugation

Bacterial intracellular folate, as well as eucaryotic folates in, *e.g* yeasts and plants, are present mostly in the form of polyglutamates. The number of glutamate residues varies commonly between 2 and 11 glutamates, and distribution depends on the organism and growth conditions (Bassett *et al.*, 1976; Sybesma *et al.*, 2003b). Three or more residues are predominant (Shane *et al.*, 1983; Sybesma *et al.*, 2003b). The current methods for folate analysis have limitations regarding polyglutamate detection. The MA using *L. casei* subsp. *rhamnosus* ATCC 7469 yields a similar response only to mono-, di-, and triglutamates, whereas response to polyglutamates with longer chains is low or absent (Eitenmiller *et al.*, 2008; Goli and Vanderslice, 1992). Most HPLC methods allow the determination of only folate monoglutamates (Eitenmiller *et al.*, 2008; Jagerstad and Jastrebova, 2013; Strandler *et al.*, 2015). Therefore, the deconjugation of folate polyglutamates is essential for reliable results in the analysis. For deconjugation, the sample may be incubated with for instance, chicken pancreas conjugase or rat serum for deconjugation of folate polyglutamates to monoglutamates, followed by boiling for a short time. Chicken pancreas deconjugates folate polyglutamates to diglutamates and can therefore be used only for MA, its pH-optimum is 7.8 (Arcot and Shrestha, 2005). Rat serum provides efficient deconjugation of folate polyglutamates to monoglutamates without compromising stability because the pH- optimum of rat plasma conjugase is 6.2-7.5 (Horne *et al.*, 1981).

Detection/quantification

Microbial folate production (e.g. expressed as the change in folate content in a microorganism per unit biomass and culturing time in a folate-free medium), as well as folate content in e.g foods, can be assessed either by microbiological assays (MA)[4-6] or high-performance liquid chromatographic (HPLC) techniques[2] coupled to detection with UV absorbance or mass spectrometric (MS) technology. Both methodologies are widely used for folate analysis and have their pros and cons.

Microbiological assay (MA). The basis for the MA is to select a microorganism that requires an external supply of folate *and* is unable to synthesize the folate by itself. Provided everything else needed is present in the medium, the growth will depend on the amount of folate in the added sample. The MA is very sensitive (sub-nanogram levels) and does not

require advanced analytical instruments, and hence often possible to perform at laboratories with microbiological skills and facilities. However, it is time-consuming and strictly quantitative and will therefore not give information on the relative concentration of different folate forms. It is critical to choose the right species and strain of microorganisms for the MA assay since it depends on equal response on growth for the different folate derivatives. Different species have been tried, including *Enterococcus hirae* ATCC 8043 or *Pediococcus acidilactici*. However, The MA using *L. casei* subsp. *rhamnosus* ATCC 7469 has shown to yield equal response to different folate forms and has hence become standard.

Different research groups have continued to develop the MA to increase reproducibility and shorten the time (from several days down to approximately one). For instance, the use of glycerol cryoprotected frozen inoculum (in contrast to stepwise precultures) of *L. rhamnosus* and applying automatic microtiter plate readers to assess the growth has reduced the cost and time of the assay[7,8].

A potential risk is that slightly different lab routines and history of the test organism may lead to inter-laboratory differences when using MA. This means that, provided good microbiological understanding and facilities for the maintenance of bacterial culture, quantification in relative terms may be very accurate between samples within one lab, but less reproducible between different labs.

HPLC. The application of HPLC separates and detects individual different folate forms but requires expensive equipment and expertise in using these techniques and interpreting the data. Ion-exchange chromatography is a popular technique for separation and purification of individual folates. Purification of extracts has been performed using different columns such as DEAE-Sephadex A-25[9]; weak anion-exchange column[10,11]; a strong anion exchanger[12] and cation-exchanger[13].

UV absorbance detection method responds to all folates, although its sensitivity to samples having low folate concentration[14] can be low. There is a good correlation, however, between the folate values detected by UV absorption and those quantified by *Lactobacillus rhamnosus* [15,16]. fluorescence detectors have also been successfully used.

Mass spectrometric (MS) detection provides new opportunities for HPLC determination of

folates because of its high selectivity and sensitivity. Different types of mass spectrometric detectors have been used for folate HPLC analysis: single quadrupole (MS), triple quadrupole or tandem mass spectrometer (MS/MS), and matrix-assisted laser desorption/ionization mass spectrometer (MALDI/MS)[17-19]. These detectors have been shown to be superior to UV- and fluorescence detectors regarding limit of determination, selectivity, and sensitivity. Recently, novel methods combining HPLC with mass spectrometric detection, such as LC-MS/MS and LC-MALDI/MS have been successfully applied to profiling of different folate derivatives in bacteria[18,20,21]. The development of these methods requires very careful optimization of sample pretreatment, especially purification steps, as well as separation on HPLC column in order to minimize the matrix effects that can result in ion suppression/enhancement of MS signal.

Bibliography

1. Hjortmo SB, Hellström AM, Andlid TA. Production of folates by yeasts in Tanzanian fermented togwa. *FEMS Yeast Res*. 2008;8(5):781-787. doi:10.1111/j.1567-1364.2008.00398.x

2. D’Aimmo MR, Mattarelli P, Biavati B, et al. The potential of bifidobacteria as a source of natural folate. *J Appl Microbiol*. 2012;112(5):975-984. doi:10.1111/j.1365-2672.2012.05261.x

3. De Brouwer V, Storozhenko S, Van De Steene JC, et al. Optimisation and validation of a liquid chromatography–tandem mass spectrometry method for folates in rice. *J Chromatogr A*. 2008;1215(1):125-132. doi:https://doi.org/10.1016/j.chroma.2008.11.004

4. Masuda M, IDE M, UTSUMI H, NIIRO T, SHIMAMURA Y, MURATA M. Production Potency of Folate, Vitamin B12, and Thiamine by Lactic Acid Bacteria Isolated from Japanese Pickles. *Biosci Biotechnol Biochem*. 2012;76(11):2061-2067. doi:10.1271/bbb.120414

5. Sybesma W, Starrenburg M, Tijsseling L, Hoefnagel MHN, Hugenholtz J. Effects of cultivation conditions on folate production by lactic acid bacteria. *Appl Environ Microbiol*. 2003;69(8):4542-4548. doi:10.1128/AEM.69.8.4542-4548.2003

6. Sugahara H, Odamaki T, Hashikura N, Abe F, Xiao JZ. Differences in folate production by bifidobacteria of different origins. *Biosci Microbiota, Food Heal*. 2015;34(4):87-93. doi:10.12938/BMFH.2015-003

7. Dang J, Arcot J, Shrestha A. Folate retention in selected processed legumes. *Food Chem*. 2000;68(3):295-298. doi:https://doi.org/10.1016/S0308-8146(99)00202-2

8. Wilson SD, Horne DW. High-performance liquid chromatographic determination of the distribution of naturally occurring folic acid derivatives in rat liver. *Anal Biochem*. 1984;142(2):529-535. doi:https://doi.org/10.1016/0003-2697(84)90502-5
9. Gregory JF 3rd, Sartain DB, Day BP. Fluorometric determination of folacin in biological materials using high performance liquid chromatography. *J Nutr*. 1984;114(2):341-353. doi:10.1093/jn/114.2.341
10. Goli DM, Vanderslice JT. Investigation of the conjugase treatment procedure in the microbiological assay of folate. *Food Chem*. 1992;43(1):57-64. doi:https://doi.org/10.1016/0308-8146(92)90242-T
11. Rebello T. Trace enrichment of biological folates on solid-phase adsorption cartridges and analysis by high-pressure liquid chromatography. *Anal Biochem*. 1987;166(1):55-64. doi:10.1016/0003-2697(87)90545-8
12. Vahteristo L, Lehikoinen K, Ollilainen V, Varo P. Application of an HPLC assay for the determination of folate derivatives in some vegetables, fruits and berries consumed in Finland. *Food Chem*. 1997;59(4):589-597. doi:https://doi.org/10.1016/S0308-8146(96)00318-4
13. Duch DS, Bowers SW, Nichol CA. Analysis of folate cofactor levels in tissues using high-performance liquid chromatography. *Anal Biochem*. 1983;130(2):385-392. doi:10.1016/0003-2697(83)90604-8
14. Gregory JF. Chemical and Nutritional Aspects of Folate Research: Analytical Procedures, Methods of Folate Synthesis, Stability, and Bioavailability of Dietary Folates. In: Kinsella JEBT-A in F and NR, ed. Vol 33. Academic Press; 1989:1-101. doi:https://doi.org/10.1016/S1043-4526(08)60126-6
15. Seyoum E, Selhub J. Combined affinity and ion pair column chromatographies for the analysis of food folate. *J Nutr Biochem*. 1993;4(8):488-494. doi:https://doi.org/10.1016/0955-2863(93)90068-8
16. Osseyi ES, Wehling RL, Albrecht JA. HPLC determination of stability and distribution of added folic acid and some endogenous folates during breadmaking. *Cereal Chem*. 2001;78(4):375-378. doi:10.1094/CCHEM.2001.78.4.375
17. Arcot J, Shrestha A. Folate: methods of analysis. *Trends Food Sci Technol*. 2005;16(6):253-266. doi:https://doi.org/10.1016/j.tifs.2005.03.013
18. Arnold RJ, Reilly JP. Observation of tetrahydrofolylpolyglutamic acid in bacteria cells by matrix-assisted laser desorption/ionization mass spectrometry. *Anal Biochem*. 2000;281(1):45-54. doi:10.1006/abio.2000.4527

19. Hjortmo S, Patring J, Jastrebova J, Andlid T. Biofortification of folates in white wheat bread by selection of yeast strain and process. *Int J Food Microbiol*. 2008;127(1-2):32-36. doi:10.1016/j.ijfoodmicro.2008.06.001
20. Leung K-Y, De Castro SCP, Cabreiro F, Gustavsson P, Copp AJ, Greene NDE. Folate metabolite profiling of different cell types and embryos suggests variation in folate one-carbon metabolism, including developmental changes in human embryonic brain. *Mol Cell Biochem*. 2013;378(1-2):229-236. doi:10.1007/s11010-013-1613-y
21. Lu W, Kwon YK, Rabinowitz JD. Isotope ratio-based profiling of microbial folates. *J Am Soc Mass Spectrom*. 2007;18(5):898-909. doi:10.1016/j.jasms.2007.01.017
